# Non-typical persistent hyperplastic primary vitreous: a rare case report and review of the literature

**DOI:** 10.1186/s12886-023-03024-x

**Published:** 2023-06-13

**Authors:** Yinhui Yu, Yue Qiao, Silong Chen, Jianghua Hu, Jinyu Li, Ke Yao, Yibo Yu

**Affiliations:** 1 Department of Eye Center, the Second Affiliated Hospital, Zhejiang University School of Medicine, Hangzhou, Zhejiang Province China; 2 Zhejiang Provincial Key Lab of Ophthalmology, Hangzhou, Zhejiang Province China; 3 Department of Ophthalmology, Jiande Branch, the Second Affiliated Hospital, Zhejiang University School of Medicine, Hangzhou, China

**Keywords:** Persistent hyperplastic primary vitreous, Age-related cataract, Diagnosis, Histopathology, Surgery

## Abstract

**Background:**

Persistent hyperplastic primary vitreous (PHPV), also known as persistent fetal vasculature (PFV), is a clinical entity that traditionally presents with leukocoria, microphthalmia, retinal dysplasia, or eyeball shrinkage which is associated with poor vision. However, there is a dearth of literature on cases of PHPV in adulthood or with asymptomatic occurrence. This report presents the clinical and pathological findings of a non-typical PHPV case and discuss the current knowledge for this condition.

**Case presentation:**

A 68-year-old healthy male was referred to our outpatient department for evaluation of age-related cataract without other visual symptoms. Preoperative fundus examination occasionally detected an isolated stalk-like band extending to the posterior pole of the eye with normal central vitreous and retina. Other ocular examinations including b-mode ultrasonography, optical coherence tomography did not unveil any abnormalities, which caused diagnostic uncertainty. We referred to cataract surgery along with histopathological study, that revealed characteristics of PHPV including fibrous connective tissues mainly composed of fibrocyte proliferation and a very few capillary vessels. Thereafter, a definitive diagnosis of non-typical PHPV was established.

**Conclusion:**

Our case is unique due to it was not discovered until adulthood, presence with only age-related cataract, and accompanied with normal central vitreous and retina. Histopathological explorations lead to an accurate diagnosis of the condition. Those results broaden the phenotype spectrums of PHPV and further provide clinical clues for the cognition of the disease.

## Background

Persistent hyperplastic primary vitreous (PHPV), also known as persistent fetal vasculature (PFV), is a rare congenital malformation of the eye resulting from continuous proliferation of the original vitreous and hyaloid vasculature, which failed to regress normally during the embryonic period [1, 2]. It is typically manifested as retrolental fibrovascular remnants or a fibrovascular stalk that extends from the optic disc to the lens to varying degrees. A variety of anterior and posterior segment abnormalities can be accompanied with PHPV, including leukocoria, microphthalmia, cataract, elongated ciliary processes, optic nerve hypoplasia, and retinal dysplasia [3, 4]. Also, it can cause serious complications such as angle-closure glaucoma, hyphema, vitreous hemorrhage, and tractional retinal detachment [5].

The knowledges of PHPV have evolved over time. Recent studies reported the involvement of various signaling pathways in the pathogenesis of PHPV, including protooncogene ski, tumor suppressor gene Arf, p53, Bax and Bak, ephrin-B2, ephrin-A5, FZD4 and LRP5 [6–10]. Besides, the existing literature provides evidence that angiogenic factors including vascular endothelial growth factor and macrophages play a role in regression of hyaloid vasculature [11]. What is more, the impaired EGFR-MTORC1-autophagy signaling may adversely affect the vascular remodeling processes essential to regression of the fetal vasculature, which can be rescued by an EGFR inhibitor of gefitinib in vivo to serve as a novel therapy for PHPV disease [12].

The heterogeneity of this disease makes diagnosis challenging. The definitive diagnoses of PHPV are typically established according to b-mode ultrasonography, color doppler ultrasound, and radiological investigations including CT and MRI imaging [3, 13, 14]. According to the literature, approximately 90% of PHPV cases are sporadic and unilateral, with no difference in incidence between males and females, and are associated with poor vision in the affected eye [2, 15, 16]. However, non-typical cases of PHPV in adulthood or those occur asymptomatically have rarely been reported, which presenting a diagnostic challenge for ophthalmologists.

In this study, we describe the case of a 68-year-old otherwise healthy male patient who complained of merely age-related cataract to our outpatient department without other visual symptoms. During preoperative routine fundus examination, a gray, stalk-like band extending peripherally into the vitreous cavity was occasionally detected. However, the results of B-scan ultrasound and other clinical examinations failed to yield a definite diagnosis. Thus, we referred to histopathological explorations of the patient’s tissue obtained during cataract surgery through which an ultimate diagnosis of atypical PHPV was established.

Herein, we will present the clinical and pathological findings of this unusual case and discuss the diagnosis and therapeutic options available for this condition. This case was unique due to it was not discovered until adulthood, as well as mild clinical characteristics. The results broaden the phenotype spectrums of PHPV and further provide clinical clues for the cognition of the disease.

## Case presentation

### Initial examination

**Fig. 1:**
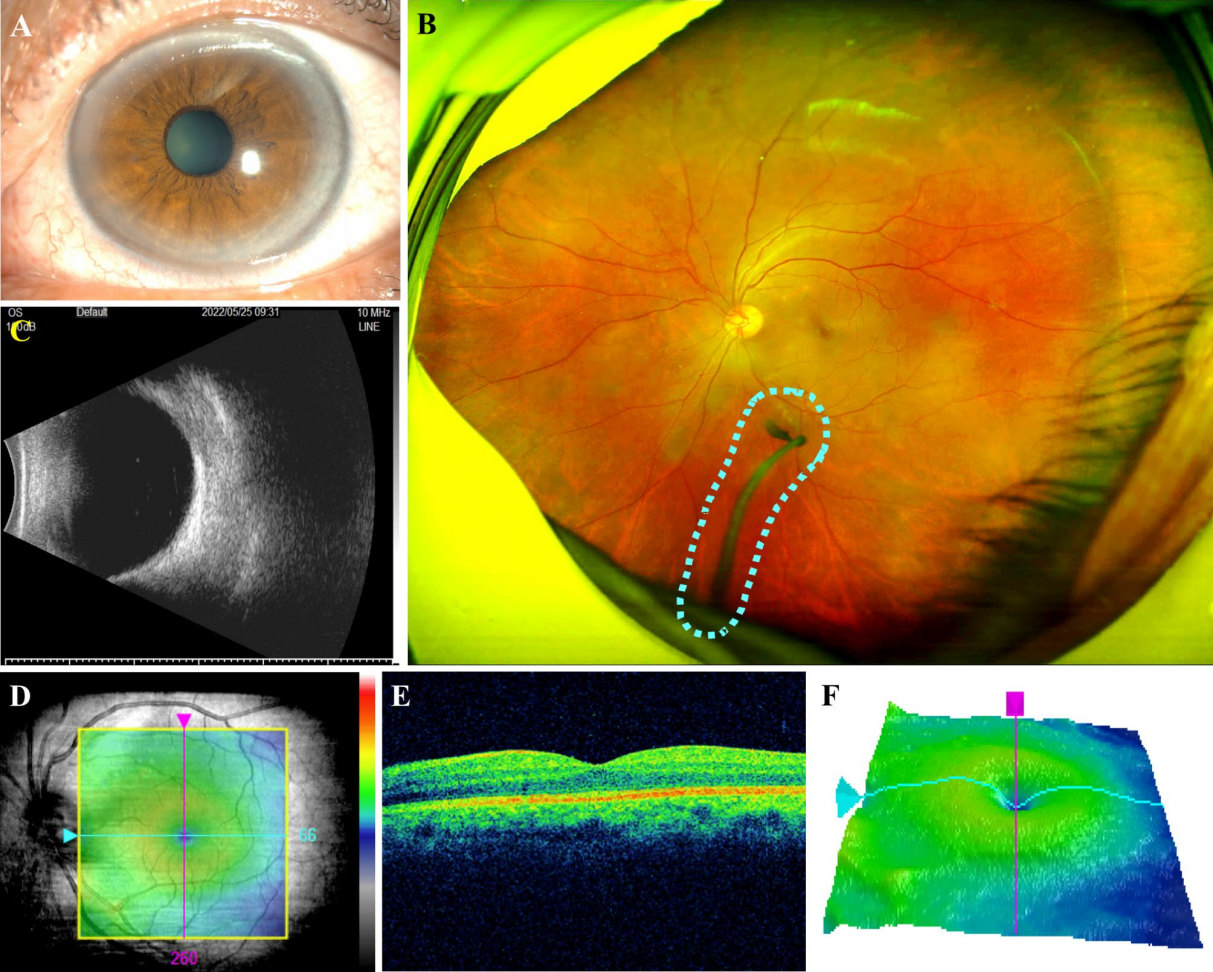
Preoperative examinations of the patient’s left eye. **A** The anterior segment photograph showed age-related cataract. **B** The digital photograph of the left fundus showed a gray, stalk-like band in the posterior segment that extended peripherally into the vitreous cavity, with normal optic disc and retina vasculature. **C** The b-scan ultrasonography showed a seemingly normal vitreous. **D-F** The optical coherence tomography (OCT) was unremarkable

A 68-year-old male presented to our outpatient department in April 2022 with a chief complaint of blurred vision in his left eye for 1 year. He denied a history of infection, trauma, and ocular surgery. There was no antenatal, postnatal, or familial history related to the disease. During the ophthalmological examination, his left eye was found to have age-related cataract with a best corrected visual acuity (BCVA) of 20/100. The cornea was clear, the anterior chamber was normal, the pupil was round and regular, and it reacted normally to light (Fig. 1A). Fundus photography of the left eye revealed an attached retina with well-shaped blood vessels, and a normal-colored optic disc. However, we detected a gray stalk-like band in the peripheral posterior segment that extended into the vitreous cavity (Fig. 1B). To elicit more evidence, b-mode ultrasonography was then conducted, however, we did not reveal any echogenic masses, stalk-like band or evidence of calcification in the vitreous and it showed a normal central hyaline cavity (Fig. 1C). Other ocular examinations also did not unveil any abnormalities, for which they revealed that the axis length of the left eye was 23.42 mm, the intraocular pressure was 14 mmHg, the corneal diameter was 11.5 × 10.5 mm, the anterior chamber depth was 2.86 mm, and the structure of the macular was generally normal (Fig. 1D-F). The contralateral eye was entirely normal with a BCVA of 20/20, and there were no systemic abnormalities or medical histories of note.

## Intraoperative observations

Surgery was indicated due to the presence of cataract. The cornea of the left eye was detected to have regular astigmatism (K1 43.1 D, K2 45.5 D, astigmatism 2.4 D) based on the corneal tomography performed with Scheimpflug imaging (Pentacam, Oculus, Germany). Consequently, the patient was subjected to a toric intraocular lens (IOL) implantation in his left eye. Preoperative marking of the toric IOL axis was conducted with the patient in an upright position to avoid misalignment due to cyclotorsion using the Robo Marker (Surgilum, USA).

**Fig. 2:**
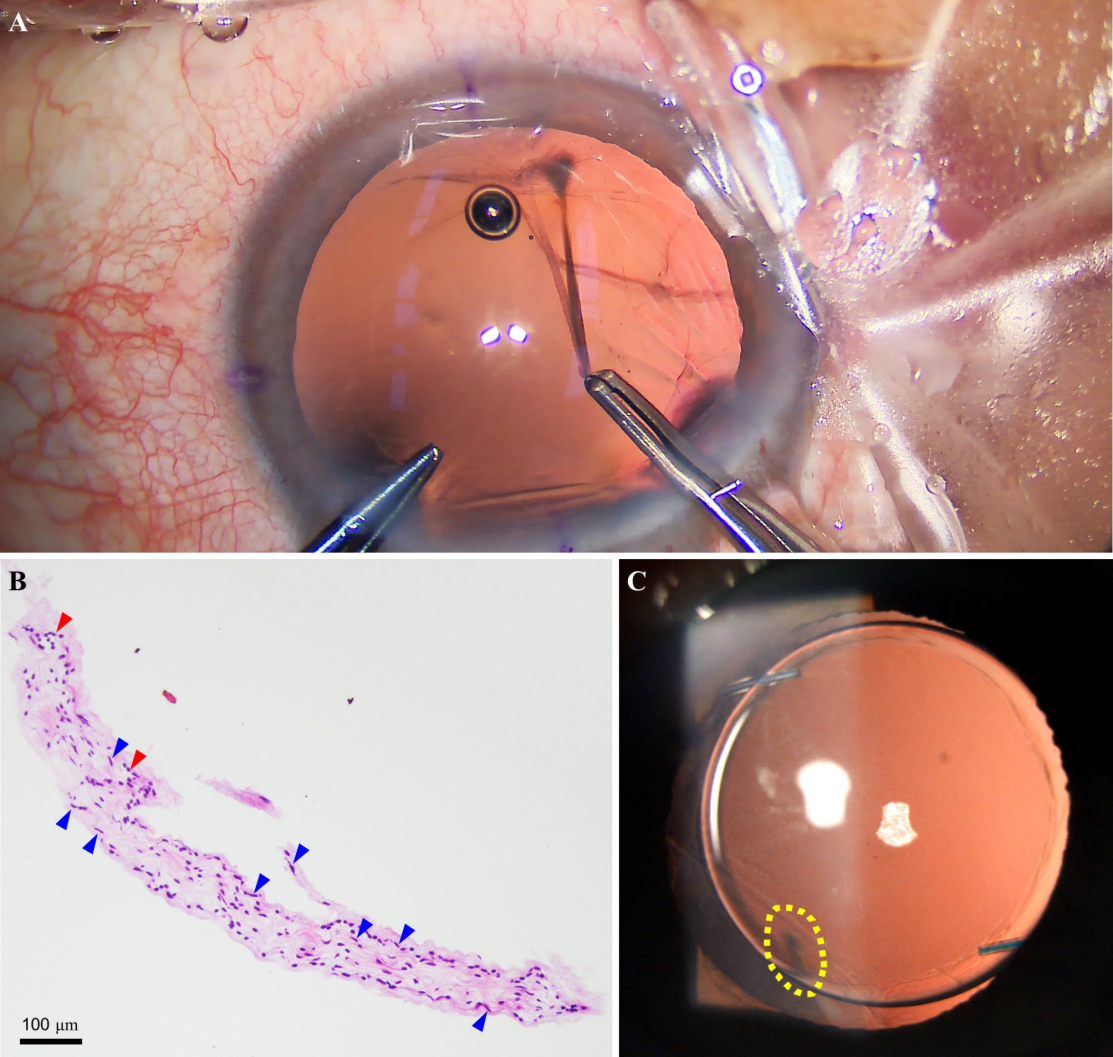
Intraoperative observations and postoperative examinations of the patient’s left eye. **A** The intraoperative photograph showed a floating fibro-vascular stalk-like band attached to the very peripheral of the posterior lens capsule, which ruptured during surgery. **B** The histological photograph of the tissue revealed fibrous connective tissues, a proliferation of fibrocytes (yellow arrows), and a few capillary vessels (red arrows). Hematoxylin and eosin. (Bar = 100 μm). **C** Slit-lamp photography showed a normal anterior segment after surgery

Intraoperatively, after removal of the lens, a floating fibro-vascular stalk-like band attached to the very peripheral, mainly at the nasal part of the posterior lens capsule and extended its free tail into the vitreous cavity was detected. The posterior capsule ruptured when the viscoelastic substance was injected into the capsular bag (Fig. 2A and see video). For the removal of the vitreous, an anterior vitrectomy was performed. The corneal incision was extended, a 20.0 Diopter IOL was implanted in the ciliary sulcus (Sensar AR40e, Abbott Medical Optics, Inc; Santa Barbara, CA), and the incision was closed with watertight suture tightening. No other intraoperative complications like hyphema, vitreous hemorrhage, glaucoma, and retinal detachment were observed during surgery.

## Histopathological findings

As the above clinical findings were not sufficient for diagnostic disclosure, we excised a small part of the tissue behind the lens using an intraocular scissor for hematoxylin and eosin (H&E) staining. Histopathological examinations demonstrated that the tissue was a fibrous connective tissue mainly composed of proliferation of fibrocytes, and a very few capillary vessels (Fig. 2B). These findings were compatible with the elements of PHPV described by Boeve et al. [17, 18]. Based on these surgical findings and histopathological results, a final diagnosis of PHPV was made.

## Follow-up visits

**Fig. 3:**
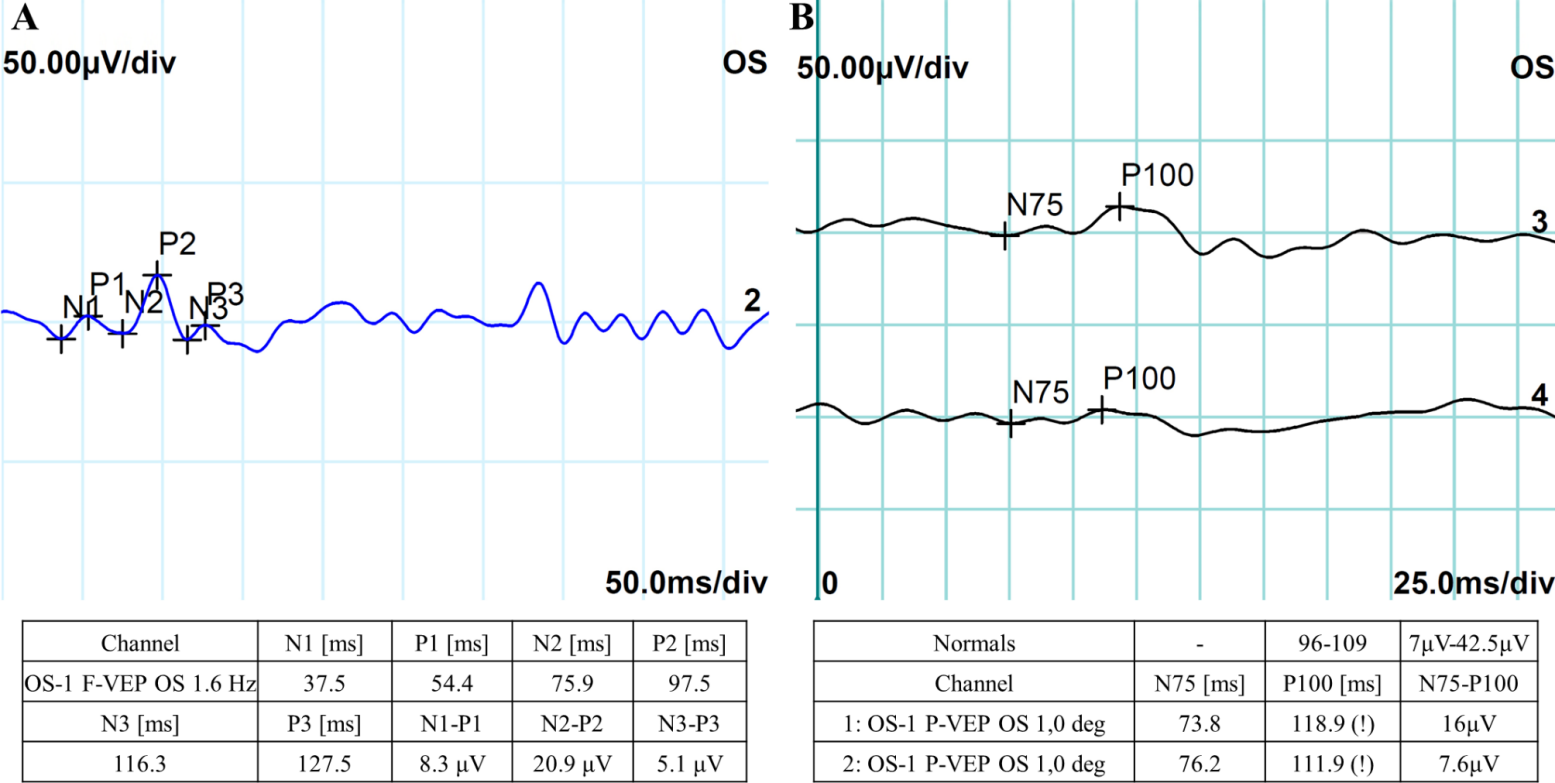
Postoperative F-VEP and P-VEP examinations of the patient’s left eye. **A** F-VEP P2 wave results showed a normal amplitude of 20.9 µV (range: 7–20 µV) and peaking time of 97.5 ms (range: 95–110 ms). **B** P-VEP P100 waveform results showed a normal amplitude of 7.6–16 µV (range: 7-42.5 µV) and a slightly delayed latency of 111.9-118.9 ms within the limits of 30% (range: 96–109 ms)

Postoperatively, the patient was treated with a standard protocol consisting of topical antibiotic and anti-inflammatory therapy agent for a duration of 2 weeks. We followed up with the patient for 5 months. The BCVA in his operated eye improved and remained in 20/20, with a transparent cornea, normal anterior chamber, a round pupil, normal IOP, and with the IOL in the proper location (Fig. 2C). To objective evaluate the visual function for the patient, we collectively performed flash visual-evoked potential (F-VEP) and pattern visual-evoked potential (P-VEP) (RETI-scan, ROLAND CONSULT, Germany) after cataract extraction. The results showed a normal amplitude of 20.9 µV (range: 7–20 µV) and peaking time of 97.5 ms (range: 95–110 ms) of FVEP P2 wave (Fig. 3A), a normal amplitude of 7.6–16 µV (range: 7-42.5 µV) and a slightly delayed latency of 111.9-118.9 ms within the limits of 30% (range: 96–109 ms) of P-VEP P100 waveform (Fig. 3B) [19]. Besides, the retina remained attached in his left fundus, and examinations in the remaining ocular posterior segments were identical to the preoperative examinations.

## Discussion and conclusions

Persistent hyperplastic primary vitreous (PHPV) is a rare congenital ocular abnormality caused by the continuous proliferation of the original vitreous in the embryonic stage without degeneration [1]. The clinical presentations in PHPV are most commonly evident in early childhood, with strabismus, microphthalmia, leukocoria, retinal dysplasia and eyeball shrinkage, and are usually associated with poor visual prognosis in the affected eye [20, 21]. However, we reported a non-typical case of PHPV, which demonstrated several unique features: (i) The patient was a previously healthy 68-year-old adult, which was very unusual as PHPV usually presents in childhood; (ii) he was asymptomatic, except for a complaint about age-related cataract and blurred vision for only 1 year, which has rarely been reported in the literature; and (iii) the fundus observation constituted only an isolated stalk-like fibrovascular band extending to the posterior pole with normal central vitreous and retina, which caused diagnostic uncertainty.

One of the unique features of our patient is that the fibrovascular stalk persists into adulthood until it is discovered. In the fetus, the original vitreous of the eye is formed in the seventh week of gestation and contains branches of the hyaloid artery between the crystalline lens and retina. During later stage, the original vitreous and hyaloid vasculature are replaced by avascular secondary vitreous, which initiates in the 20th week of gestation and completes at birth [3]. As has been reported, the failure of these primary vitreous and vessels to regress completely can lead to PHPV [22]. The isolated fibrovascular stalk in our case suggests an uncomplete regression course. We referred to the pathogenesis of PHPV in the literature, it has been reported that a number of genes and pathways primarily associated with eye morphogenesis, the apoptotic process, autophagy, and the angiogenic process were involved in the regression of hyaloid vasculature [23]. Besides, the macrophages may also play a central role in the blocking of blood flow, induction of apoptosis, and clearance of atrophic vessels during regression of the hyaloid vascular system [24, 25]. However, there were no systemic abnormalities of note in our patient, which suggested a somewhat unusual course of hyaloid vasculature regression.

Another unusual feature is that our case constituted only an isolated stalk-like fibrovascular band extending to the posterior pole combined with normal central vitreous and retina. Based previous report, the vast majority of PHPV cases have a combination of anterior and posterior segment involvement [5]. Anterior PHPV manifested as the presence of a retrolental fibrovascular membrane, which gradually covers the posterior surface of the lens, grows into the lens itself, invades the ciliary process, and leads to various complications, including posterior polar cataract, secondary glaucoma, iris vascular remnants, or intra-lenticular hemorrhage [20, 26]. Posterior PHPV is presented with an elevated vitreous membrane or stalk extending from the optic disc to the peripheral retina or retrolental region, along with abnormalities involving the posterior segment, such as retinal folds, macula dysplasia, optic nerve hypoplasia, or retinal detachment [2]. While in typical case of PHPV, both anterior and posterior signs can be observed. In the present case, the characteristics of anterior PHPV, like retrolental opacity, elongated ciliary processes, leukocoria, or angle-closure glaucoma, were not observed. The presence of an elevated fibrovascular stalk in the vitreous cavity may fall under the posterior type of PHPV, but the membrane did not connect the optic disc or alter the structure of the posterior segment. Moreover, typical signs of posterior PHPV, such as anomaly of the optic disc, macula, or retina, were also not observed in the affected eye in our patient. All things being considered, it was difficult to categorize disclosure based on conventional presentations.

Thirdly, one more rare feature of the current case is that the patient is asymptomatic and denied any vision impairment in early childhood. Predictors of poor visual prognosis in PHPV include bilaterality, microphthalmos, glaucoma, and posterior segment involvement, which will lead to an increased risk of retinal detachment or even eyeball shrinkage. Posterior PHPV usually leads to a poor vision prognosis, according to previous studies [4, 5]. The degree of ocular malformation will ultimately determine the visual outcome. In our case, the patient did not involve in any of the above complications that could have resulted in a poor vision outcome. In addition, electrophysiological testing of VEPs, currently the only non-invasive, objective method for evaluating neural function in the pathway between retinal ganglion cells and the visual cortex [27], were in normal range, which suggested the disease did not invade the optic nerve or affect the neuron function. Consequently, a normal functional integrity of the visual system and a satisfied vision rehabilitation were achieved in our patient.

Clinically, the diagnosis of PHPV is usually made based on typical clinical signs and imaging manifestations. It is now accepted that the b-scan ultrasonography, color doppler ultrasound, CT, and MRI are vital for disease diagnosis, especially in eyes in which the posterior segment is not visible [3, 28]. The b-scan ultrasound can show the stalk going from the lens to the posterior pole, moreover, the color-flow doppler can show the vascular nature of PHPV lesions [13]. CT scanning can also show the PHPV membrane and the absence of calcification which is useful in the diagnosis of PHPV as well as differentiating PHPV from retinoblastoma. Furthermore, the morphology of these various lesions can be seen clearer with MRI [14]. In our case, b-mode ultrasonography did not reveal any echogenic masses, stalk-like band or evidence of calcification in the vitreous and it showed a normal central hyaline cavity. We speculated this may be caused by the inspector’s failure to operate at a proper angle or neglect. For this non-typical case, we conduct histopathological examinations in which fibrous connective tissues composed of a proliferation of fibrocytes, and a very few capillary vessels in the excised tissue was revealed. Accordingly, a final diagnosis of non-typical PHPV was established.

Surgery was indicated for the patient due to the presence of cataract. During surgery, the fibrovascular stalk was observed to be connected to the posterior lens capsule, which ruptured due to the dragging of the fibrous membrane. This is a known complication of PHPV and is often ascribed to the invasion of the abnormal fibrovascular tissue which exerted increased traction on the posterior capsule [29]. As a result, the peripheral posterior capsule might have been weakened by the fibrovascular tissue, which are more vulnerable to being ruptured. In fact, previous studies on retrolental remnants of PHPV have also revealed early disturbance of lens development and a failure to form posterior lens fibers, which finally led to development defects of the posterior capsule [3]. In the present case, a combined anterior vitrectomy with IOL implantation in the ciliary sulcus was undertaken subsequently. The BCVA in his operated eye improved and remained in 20/20, with a transparent cornea, normal anterior chamber, a round pupil, normal IOP, and with the IOL in the proper location. This also proved that our treatment was effective and safe.

In conclusion, we reported a non-typical case of PHPV in a 68-year-old adult, he was previously healthy, presented with only age-related cataract, and accompanied with normal central vitreous and retina. Our case broadens the phenotype spectrums of PHPV and provides clinical clues for the cognition of the disease. More importantly, we successfully diagnosed this non-typical PHPV case with a histological approach. We suggest that a similar approach may be considered for this patient group.

## Data Availability

The original data presented in the study are included in the article, without undue reservation. Further inquiries can be directed to the corresponding author.

## Abbreviations

PHPV: Persistent hyperplastic primary vitreous
PFV: Persistent fetal vasculature
BCVA: Best corrected visual acuity
IOL: Intraocular lens
H&E: Hematoxylin and eosin
F-VEP: Flash visual-evoked potential
P-VEP: Pattern visual-evoked potential
OCT: Optical coherence tomography
